# Biological roles of CCAAT/Enhancer-binding protein delta during inflammation

**DOI:** 10.1186/s12929-014-0110-2

**Published:** 2015-01-16

**Authors:** Chiung-Yuan Ko, Wen-Chang Chang, Ju-Ming Wang

**Affiliations:** Program for Neural Regenerative Medicine, College of Medical Science and Technology, Taipei Medical University, Taipei, 11031 Taiwan; Center for Neurotrauma and Neuroregeneration, Taipei Medical University, Taipei, 11031 Taiwan; Graduate Institute of Medical Sciences, College of Medicine, Taipei Medical University, Taipei, 11031 Taiwan; Institute of Bioinformatics and Biosignal Transduction, College of Bioscience and Biotechnology, National Cheng Kung University, Tainan, 70101 Taiwan; Infectious Disease and Signaling Research Center, National Cheng Kung University, Tainan, 70101 Taiwan; Center of Molecular Inflammation, National Cheng Kung University, Tainan, 70101 Taiwan

## Abstract

CCAAT/enhancer-binding protein delta (CEBPD) belongs to the CCAAT/enhancer-binding protein family, and these proteins function as transcription factors in many biological processes, including cell differentiation, motility, growth arrest, proliferation, cell death, metabolism and immune responses. The functional diversity of CEBPD depends, in part, on the cell type and cellular context, which indicates that CEBPD could interpret a variety of cues to adjust cellular responses in specific situations. Here, we review the regulation of the CEBPD gene and its function in response to inflammatory stimuli. We also address its effects in inflammation-related diseases through a discussion of its recently discovered downstream targets. Regarding to the previous discoveries and new insights in inflammation-associated diseases, suggesting CEBPD could also be a central gene in inflammation. Importantly, the results of this study indicate that the investigation of CEBPD could open a new avenue to help better understand the inflammatory response.

## Introduction

CCAAT/enhancer-binding protein delta (CEBPD) is an intronless gene that encodes a 269-amino acid protein belonging to the CCAAT/enhancer-binding protein family of proteins, which are known to function as transcription factors in cellular differentiation [1-3], metabolism [4] and immune responses [5]. At present, six C/EBP members have been identified in humans: CEBPA (C/EBP, RcC/EBP-1), CEBPB (NF-IL6, LAP, CRP2, NF-M), CEBPG (Ig/EBP-1), CEBPD (NF-IL6β, CRP3, CELF, RcC/EBP2), CEBPE (CRP-1) and CEBPZ (CHOP-10, GADD153). All of the members of this family contain very diverse N-termini, a highly conserved (>90% identity) basic leucine zipper domain and a basic domain for dimerization and DNA binding at their C termini [6]. Moreover, several posttranslational modifications, including acetylation, sumoylation and phosphorylation were also identified on C/EBP family members. Therefore, the highly conserved DNA binding domains explain how C/EBP family members can recognize similar DNA sequences in *in vitro* and *in vivo* DNA binding assays [7-10] but show diverse functions in cells, which may result from interaction with various proteins involving in transcriptional regulation or post-translational modifications.

## Review

### Regulation of CEBPD expression during inflammation

The expression level of CEBPD is typically low in most cells at normal physiological conditions but is rapidly induced by external stimuli including glucocorticoids [11-13], insulin [8,14,15] and growth factors, e.g., epidermal growth factor [16]. In inflammatory environments, CEBPD has been suggested to be activated by inflammatory factors, such as interleukin-6 (IL-6) [17,18], lipopolysaccharides (LPS) [19-25], interferon-α, interferon-γ (IFN-γ) [26], tumor necrosis factor-α (TNFα) [27-30], prostaglandin E2 (PGE2) [31-33] and interleukin-1β (IL-1β) [6,29,30,34,35]. Although the above studies suggested that CEBPD contributes to proinflammation, a study demonstrated that CEBPD mediates in IFN-γ- and IL-1β- induced anti-apoptosis and anti-inflammation in pancreatic β-cells [36]. However, the details including signaling pathways and responsive transcription factors in response to the above stimuli to regulate the activation of *CEBPD* gene remain less characterized.

In response to inflammatory stimuli, *CEBPD* gene transcription can be activated through the phosphatidylinositol 3-kinase [37], p38 [16,30], JAK [18], JNK [21] and PKA [31] signaling pathways. Interestingly, in addition to transcriptional activation through the *CEBPD* promoter, a recent study suggested that the abundance of CEBPD can be regulated at both posttranscriptional and posttranslational levels. In macrophages, nuclear HuR, an RNA-binding protein, responds to PGE2 and is shuttled to the cytoplasm to stabilize *CEBPD* mRNA [32]. In addition, the E3 ubiquitin ligase, seven in absentia homolog 2 (SIAH2), was suggested to be involved in posttranslational regulation through the polyubiquitination of CEBPD and downstream degradation via the proteasome in breast cancer cells [38]. However, it is still unclear whether this SIAH2-mediated degradation of CEBPD commonly occurs in inflamed cells.

### Role of CEBPD in cyclooxygenase 2 (COX-2) regulation

Prostaglandins play important roles in many biological processes, including cell division, immune responses, blood pressure regulation, ovulation, bone development and wound healing [39]. Prostaglandin-endoperoxide synthase 2, also known as COX-2, is an inducible enzyme whose expression is mainly regulated at the transcription level and can be induced by cytokines, growth factors, phorbol esters and endotoxins. In the past decade, tremendous progress has been made in understanding the functional roles of COX-2 in cell growth, cell death, cell motility and tumorigenesis. C/EBP family members have been suggested to be involved in regulating *COX-2* transcription through direct binding to the promoter region of the COX-2 gene. CEBPB and CEBPD bind to the *COX-2* promoter in chondrocytes following IL-1β treatment [40]. Moreover, mouse CEBPD (Cebpd) is involved in LPS- and proteasome inhibitor-induced *COX-2* gene expression in murine RAW 264.7 macrophages and alveolar epithelial cells, respectively [41,42]. Interestingly, prostaglandin E2 (PGE2), a COX-2 enzymatic product, can induce *CEBPD* gene expression [32] (Wang JM *et al.,* unpublished results). These results suggested that the regulation of COX-2 and CEBPD could be functioning in a positive-feedback loop. On the other hand, the activation of p38 mitogen-activated protein kinase (MAPK) has been observed in response to IL-1β or TNFα treatment [43-45] and has been suggested to be involved in the activation of *CEBPD* transcription [46,47]. However, whether p38-mediated CEBPD activation commonly participates in *COX-2* gene regulation in inflamed cells remains an open question and needs to be further clarified.

In our previous study, we also showed that the post-translational modification of CEBPD is involved in COX-2 regulation. The sumoylated-LAP1 (CEBPB variant) and CEBPD both act as mediators in modulating HDAC4 recruitment on the COX-2 promoter. Upon EGF treatment, the instantly induced nonsumoylated CEBPD can be a positive regulator that replaces the sumoylated-LAP1 and CEBPD. This replacement will increase the p300 binding and form an intact initiation of the activation complex to stimulate *COX-2* transcription [9]. These studies indicated that the post-translational modifications of CEBPD protein also responsible for CEBPD-mediated transcriptional regulation. In addition, the histone deacetylase inhibitor, trichostatin A, has a positive effect on LPS-induced *COX-2* transcription. It was suggested to be regulated by increasing the recruitment of CEBPA and CEBPB, but not CEBPD, to the *COX-2* promoter because the CEBPD expression was attenuated [48].

### The roles of CEBPD during acute inflammation

Activation of CEBPD has been observed in several acute inflammation diseases. However, the detailed mechanisms and regulations involving CEBPD need to be studied extensively. The acute-phase response (APR) protein takes part in the early and nonspecific (but highly complex) reaction of an organism in response to a variety of injuries, such as bacterial infection, tissue injury, extensive bleeding, LPS, turpentine oil, heavy metals, thermal injury or surgery [19,49,50]. The liver has been believed to play an important role in the production of APR during the early stages of acute inflammation. CEBPA, CEBPB and CEBPD were reported to regulate APR expression in liver cells [17,51]. Poli *et al.* demonstrated that the CEBPs involved in the induction of APR genes are linked to newly synthesized, NF-κB- and STAT3-regulated CEBPB and CEBPD proteins [35]. Moreover, phosphorylated CEBPD mediates serum amyloid A gene expression in APR-induced inflammation in rabbit liver [52].

NF-κB has long been considered to be involved in the prototypical proinflammatory response that is largely based on the role of NF-κB in the induction of proinflammatory cytokines, chemokines and adhesion molecules. CEBPD can crosstalk with NF-κB in inflamed and immune cells. In Toll-like receptor 4 (TLR4)-induced macrophage activation, NF-κB binds to the *CEBPD* promoter and activates *CEBPD* transcription. Then, CEBPD binds to the *IL-6* promoter and cooperates with NF-κB to fully activate *IL-6* transcription [53]. CEBPD also can act as an amplifier of NF-κB-mediated transcription, which discriminates transient and persistent TLR4 signals, thus facilitating the sustained expression of the inflammatory response [53]. In addition, a recent study showed that CEBPD activates TLR4 gene expression in macrophages and a CEBPD downstream target, F-box and WD repeat domain containing protein 7 alpha, would feedback negatively and downregulate CEBPD to attenuate TLR4 inflammatory signaling [22]. These discoveries suggest that TLR4 and CEBPD could form a regulatory loop in macrophages.

In addition to the classical NF-κB and MAPK pathways, LPS was shown to activate IκB kinase ε, leading to the phosphorylation and activation of CEBPD [23]. However, it has also been reported that low-dose LPS fails to activate the classical NF-κB pathway and contributes to the leaky and mild elevation of proinflammatory mediators. Selectively removing the suppressive nuclear receptors, including peroxisome proliferator-activated receptor α and retinoic acid receptor, on the promoter regions of proinflammatory mediators activates CEBPD in an interleukin-1 receptor-associated kinase 1-dependent manner [24]. As a critical mediator of LPS-induced acute lung injury, CEBPD can be repressed by the transcription factor Miz1 through recruiting the histone deacetylase HDAC1, further contributing to the suppression of inflammation [54,55]. Meanwhile, suppressor of cytokine signaling 3, a negative regulator of IL-6 signaling, has a protective role in LPS-induced acute lung injury by suppressing CEBPD activity [56]. CEBPD-dependent macrophage mobilization also plays a key role in contributing to the host’s defense against bacterial infection [57] and exaggerates bacterial dissemination through platelet-activating factor receptor-dependent bacterial translocation in pulmonary infection [58]. In addition, *Cebpb*- or *Cebpd*-deficient macrophages showed impaired induction of IL-6 and TNFα stimulated by several TLR ligands [54,59,60]. Furthermore, both CEBPB and CEBPD are key transcription factors that regulate the Fcγ receptor-mediated induction of TNFα, macrophage inflammatory protein 2 and 1α in macrophages [61]. Induction of disseminated intravascular coagulation in CEBPD-deficient mice decreased endotoxin-induced systemic inflammation when compared with wild-type mice, as evident from the decreased plasma levels of tumor necrosis factor-alpha and IL-6 [62]. On the other hand, CEBPD mediated the activation of the LPS-induced anti-inflammatory factor interleukin 10 in mouse macrophages [21]. Our results showed that CEBPD expression can be induced in both M1 and M2 macrophages (Wang JM *et al.* unpublished results). These findings implied that CEBPD could interact with different components in response to various inflammatory cytokines and plays opposite roles in initiating and resolving the inflammatory response. Interestingly, miR let-7c, which targets CEBPD, has been clarified in promoting M1 macrophage polarization and diminishing M2 phenotype expression [25]. This study further noted the possibility that CEBPD could play a regulator to switch the M1/M2 population during inflammation. Taken together, the above discoveries and controversial observations imply that CEBPD possesses a dual role in immunity/inflammation reactions and indicates that the dissection and interpretation of CEBPD’s function during inflammation is complex.

### CEBPD during neuroinflammation and inflammation-related diseases

Despite the large amount of studies that have been conducted and the solutions that have been proposed for clinical applications in chronic inflammatory diseases, our knowledge base is still not sufficient to effectively treat these diseases. Thus, the identification and investigation of novel or less-characterized, inflammation-related genes are important. The activation of CEBPD has been observed in many autoimmune and age-associated chronic inflammation diseases, such as atherosclerosis [63], type 2 diabetes [64], rheumatoid arthritis (RA) [65], Alzheimer’s disease (AD) [66] and Parkinson’s disease [67]. Recently, a more detailed involvement of CEBPD in some of these inflammation-related diseases, and even in the tumor microenvironment, has been elucidated.

#### CEBPD in AD

Though the increased levels of CEBPA and CEBPB at sites of neuroinflammation or neural damage have been mentioned [68], the identification and function of CEBP-mediated transcriptional regulation and the target genes remain rarely investigated. An immunohistochemistry assay revealed that the location of CEBPD is consistent with the cytokine-laden milieu of the AD neocortex and limbic cortex, and CEBPD is qualitatively more abundant and stains intensely in astrocytes of AD patients [66]. This observation raised several interesting questions, including whether the impairment in memory and cognition as well as the personality change, all of which are characteristics of AD patients, are due to the increased levels of CEBPD in astrocytes.

**Figure 1:**
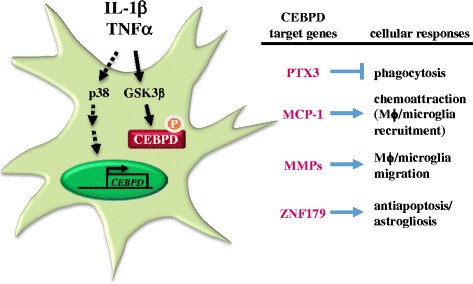
**Functional roles of astrocytic CEBPD.**

Activated phagocytic microglia also interact with astrocytes and neural cells to fight off infections as quickly as possible with minimal damage to healthy brain cells. In AD, phagocytic microglia travel to the site of injury in the inflamed brain to engulf the offending materials. We found that astrocytic CEBPD has the ability to attenuate macrophage-mediated phagocytosis of damaged neurons. One CEBPD target, pentraxin-3 (PTX3, also known as TNFAIP5 or TSG-14), participates in the attenuation of macrophage-mediated phagocytosis of damaged neurons [29]. These results provided the first molecular evidence indicating that astrocytic CEBPD and CEBPD-regulated PTX3 play a role in the accumulation of damaged neurons and repair of brain damage. It was also suggested that the detection of PTX3 in cerebrospinal fluid could be used as a diagnostic tool for AD progression. Moreover, CEBPD also regulates the chemoattractive factor gene monocyte chemotactic protein-1 (MCP-1) and the migration-promoting genes matrix metalloproteinase (MMP)-1 and MMP-3. Once astrocytic CEBPD is induced by IL-1β, the recovered GSK3β activity phosphorylates CEBPD at serine 167 (ser167), which then promotes the migration and activation of microglia/macrophages [30] through MCP-1 and MMP-1, respectively. Meanwhile, the use of the GSK3β inhibitor LiCl in this study suggested that the effect of LiCl in the clinic may not only act on neural cells but could also result from the inhibition of CEBPD in astrocytes. Furthermore, an increase in apoptotic astrocytes was observed in AppTg/*Cebpd*^−/−^ mice [an Alzheimer disease mouse model (APPswe/PS1/E9 bigenic) crossed with *Cebpd*-deficient mice], thereby suggesting that CEBPD plays a functional role in contributing to the anti-apoptotic ability of astrocytes [69]. The Zinc Finger Protein 179 (ZNF179) gene is activated by CEBPD and mediates CEBPD-induced anti-apoptosis in astrocytes by collaborating with the transcription repressor PLZF to inactivate the expression of the proapoptotic genes insulin-like growth factor-binding protein 3 and BCL2-interacting killer [69]. The biological function of CEBPD and its downstream target genes in astrocytes are indicated in Figure 1.

#### CEBPD in Traumatic brain injury (TBI)

TBI is also known to increase the expression of amyloid precursor protein (APP) [70]. Many studies have reported that TBI-induced Aβ accumulation can further contribute to AD formation [71,72], but the detailed mechanisms of TBI progression and its association with AD formation remain less characterized. CEBPD has been reported to be upregulated in the cortex of TBI rats [73]. As mentioned above, Ko *et al.* showed that astrocytic CEBPD levels increase in response to Aβ and proinflammatory factors [29]. Therefore, CEBPD could be the link between TBI-induced Aβ accumulation and AD pathogenesis. However, this speculation requires further study. In addition, inducible nitric oxide synthase (iNOS) deficiency prevented beta-amyloid deposition, astrocytosis, microgliosis and disease progression in APPTg mice [74]. CEBPD also upregulates neurotoxic iNOS expression in glial cells [75]. In *Cebpd*^−/−^ mice, systemic LPS-induced brain expression of iNOS, TNFα, IL-1β and IL-6 was attenuated [76]. These results demonstrated that CEBPD plays a crucial role in regulating proinflammatory gene expression in glial activation. However, the detailed mechanisms and the *in vivo* relevance of the roles of glial CEBPD and iNOS in neuroinflammation diseases, including TBI, remain unclear.

#### CEBPD in RA

Coincident with the increased levels of serum C-reactive protein and synovial IL-6, increased CEBPB and CEBPD expression was detected in synovial tissue of patients with RA [65]. Macrophages have been suggested to play a central immunoreactive role in RA [77,78] due to their prominent numbers at the cartilage-pannus junction. In RA, CEBPD expression was mainly observed in macrophages [65] and compared with WT mice, reduced pannus formation and greater joint architecture integrity were observed in the paws of collagen-induced arthritis *Cebpd*-deficient mice [79]. Using cytokine array and microarray analyses, we identified several secretory factors, including chemokine (C-C motif) ligand 20 (CCL20), chemokine (C-X-C motif) ligand 1 (CXCL1), IL-23A and tumor necrosis factor alpha-induced protein 6 (TNFAIP6), that were directly regulated by CEBPD in macrophages [79]. A loss of function assay showed that CCL20, IL23A, CXCL1 and TNFAIP6 contributed to the migration and proliferation of synoviocytes, but only the latter two proteins were involved in the tube formation of endothelial cells [79]. Interestingly, two potent anti-inflammatory molecules, inotilone and rosmanol [80,81], which are putative CEBPD inhibitors, were shown to inhibit the migration and proliferation of rat fibroblast-like synoviocytes as well as the tube formation of HUVECs [79]. Taken together, the elucidation of CEBPD biology and its downstream effectors could be helpful in the diagnosis of RA, and these factors could potentially serve as therapeutic targets for RA therapy.

#### CEBPD in multiple sclerosis (MS)

As an autoimmune inflammatory disease, MS is the prototypical inflammatory demyelinating disease of the central nervous system (CNS) and causes demyelination and variable axonal loss. It is characterized by multiple lesions (plaques) involving the brain and spinal cord and has an unpredictable clinical course. Experimental autoimmune encephalomyelitis (EAE) is the most commonly used experimental model for the human inflammatory demyelinating disease, MS. Mice deficient in Cebpd expression exhibited less severe clinical disease than wild-type littermates in response to induction of EAE by vaccination with a myelin oligodendrocyte glycoprotein fragment [82]. This study suggested that CEBPD suppresses expression of IL-10 in dendritic cells (DC), favoring Th17 over Treg development. These findings indicated that CEBPD plays a functional role in both DC and CNS autoimmune inflammatory disease via altering the Th17:Treg balance in an IL-10 dependent fashion.

### The role of CEBPD in macrophages of the tumor microenvironment

Epidemiological studies and animal experiments have suggested that inflammation can increase the risk of normal cells developing into tumorigenic cells as well as enhance cancer cell invasion and metastasis. Moreover, the microenvironment of solid tumors is characterized by a reactive stroma containing an abundance of inflammatory mediators, leukocytes, dysregulated vessels and proteolytic enzymes. However, the communication between cancer cells and the surrounding stromal cells, such as macrophages and fibroblasts, remains largely unclear, especially in an inflammation condition.

Tumor-associated macrophages (TAMs), a type of stromal cell, play a crucial role in promoting the progression of certain cancers. TAMs have an M2 phenotype and function in the promotion of tumor cell proliferation, angiogenesis and constitutive matrix turnover as well as in the repression of adaptive immunity [83,84]. The tumor-promoting properties of macrophages have been further revealed due to their ability to synthesize and secrete inhibitory factors that suppress anti-tumor effectors of the host. COX-2 is commonly activated in cancer cells, resulting in the production of PGE2, which exerts strong immunosuppressive effects on cell-mediated immune mechanisms [85,86].

One study demonstrated that *Cebpd*^−/−^*/HER2/neu* mice exhibit increased mammary tumor multiplicity and decreased lung metastasis [87]. The study agreed with the speculation that CEBPD acts as a tumor suppressor in cancer cells [88]. In addition, the hypoxia environment in cancer can induce CEBPD expression and contributes to the metastasis and invasion of cancer cells [87]. Actually, the decreased lung metastasis may also implied that the existence of CEBPD in stromal cells, such as fibroblasts or macrophages, in tumor microenvironment might contribute to invasion/metastasis. We found that CEBPD regulates or co-regulates a wide range of inflammatory factors, such as TNFα, IL-1β, IL-6, CXCL1, IL-17A [79], and the chemokines, monocyte chemoattractant protein 1 (MCP1) and IL-10 [30,32,89]. Therefore, several interesting and valuable issues were raised and should be addressed including (I) an investigation of the regulation of CEBPD expression in the cells surrounding a tumor, such as TAMs, and (II) a dissection of the contributions of CEBPD and the consequent effects of its activation within the tumor microenvironment.

**Figure 2:**
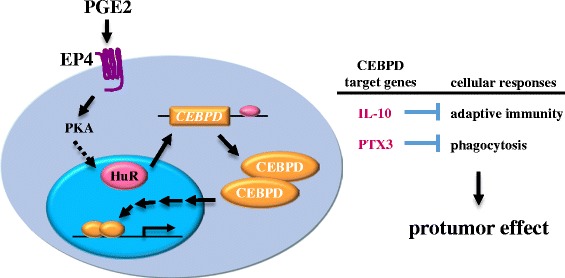
**CEBPD in tumor-associated macrophages (M2 Mϕ).**

As mentioned above, CEBPD directly regulates *COX-2* gene expression [46]. We also demonstrated that PGE2 positively regulated CEBPD expression through HuR-mediated posttranscriptional regulation in macrophages. Moreover, a cancer cell allograft mouse model provided evidence to support the idea that CEBPD plays a role in cancer growth in the protumor microenvironment [32]. Importantly, in TAMs, the CEBPD downstream target PTX3 and IL-10 were shown to suppress the ability of macrophages to phagocytose cancer cells and provide immunosuppressive effect, respectively [32]. The functional roles of PGE2-induced CEBPD in macrophages of the tumor microenvironment are shown in Figure 2. This study successfully revealed a new insight of CEBPD in the communication between cancer cells and the tumor microenvironment, especially in macrophages. Moreover, the results of this study also implied that the dissection of CEBPD’s roles in the tumor microenvironment could provide a great opportunity to develop a feasible translational application for cancer therapy.

## Conclusion

In this review, we highlighted the role of CEBPD during inflammation and inflammation-related diseases, including cancers. Recent studies have partly revealed the roles of CEBPD in inflammation and have uncovered much about CEBPD biology, especially in various cell types; however, the effects of these roles and their crosstalk between different cell types remain largely unknown. In addition, we know that posttranslational modification of proteins plays an important role in the precise function and correct response of cells to external stimuli; however, how these posttranslational modifications affect the function of CEBPD or how specific proteins interact with CEBPD and affect CEBPD’s roles in cell-type-specific manners is still unknown. CEBPD has been linked to many cellular functions and inflammatory diseases. Therefore, new insights into CEBPD’s involvement and regulation will be helpful to better understand the complexity and diversity of inflammatory processes and will also identify targets for the development of therapeutic drugs and strategies to overcome inflammation-related diseases involving CEBPD.
